# ECLAMC Study: prevalence patterns of hypospadias in South America: multi-national analysis over a 24-year period

**DOI:** 10.1590/S1677-5538.IBJU.2016.0002

**Published:** 2017

**Authors:** Nicolás Fernández, Jaime Pérez, Pedro Monterrey, Fernando A. Poletta, Darius J. Bägli, Armando J. Lorenzo, Ignacio Zarante

**Affiliations:** 1 Departamento de Urología, Pontificia Universidad Javeriana, Departamento de Urología, Hospital Universitario San Ignacio, Bogotá, Colombia;; 2 Instituto de Genética Humana, Pontificia Universidad Javeriana, Bogotá, Colombia;; 3 Departamento de Matemáticas, Rosario University, Bogotá, Colombia;; 4 ECLAMC (Estudio Colaborativo Latinoamericano de Malformaciones Congénitas) at Centro de Educación Médica e Investigaciones Clínicas (CEMIC-CONICET), Buenos Aires, Argentina and Instituto Nacional de Genética Médica Populacional (INaGeMP), Rio de Janeiro, Brasil; 5 Division of Urology, Department of Surgery, Hospital for Sick Children and University of Toronto, Toronto, Ontario

**Keywords:** Hypospadias, Prevalence, Epidemiology

## Abstract

**Objective:**

To evaluate prevalence trends of hypospadias in South-America it is essential to perform multicenter and multinational studies with the same methodology. Herein we present systematic data as part of an international multicenter initiative evaluating congenital malformations in South America over a 24-year period.

**Materials and Methods:**

A nested case-control study was conducted using the Latin American Collaborative Study of Congenital Malformations (ECLAMC), between January 1989 and December 2012. Cases were stratified as isolated (IH) and non-isolated hypospadias (NIH). Global prevalence was calculated and discriminated by country. Associations between birth weight and gestational age, and NIH distribution by associated abnormality and severity of hypospadias, were analyzed.

**Results:**

A total of 159 hospitals from six countries participated, reporting surveillance on 4.020.384 newborns. A total of 4.537 hypospadias cases were detected, with a global prevalence of 11.3/10.000 newborns. Trend analyses showed in Chile, Brazil and Uruguay a statistically significant increase in prevalence. Analysis of severity and associated anomalies did not to find an association for distal cases, but did for proximal (RR=1.64 [95% CI=1.33-2.03]).

**Conclusion:**

This is one of only a few Latin American multicenter studies reporting on the epidemiology of hypospadias in South America in the last two decades. Our data adds to evidence suggesting an increase in some countries in the region at different times. There were also variations in prevalence according to severity. This study adds to literature describing associated anomalies at a hospital-based level.

## INTRODUCTION

Despite being one of the most common congenital anomalies of the male external genitalia, after decades of research we still lack knowledge on the exact pathophysiology of hypospadias (1-3). In this regard, multiple authors have identified an increase in prevalence, information generated from studies in Europe and North America (4-6). Although a global phenomenon is plausible, there is a paucity of information on the trends and impact of this condition in many other parts of the world (7, 8).

To date, there have been few hospital-based studies with regional information including children from other parts of the World, such as Central and South America and Africa (1, 2, 9). To address this, herein we present data gathered in a systematic fashion as part of an international multi-center initiative evaluating congenital malformations in South America. We hypothesized that prevalence patterns should follow similar patterns to those presented in previous publications in other regions around the world. The aim of the present study was to analyze trends and conduct an epidemiologic description over a 24-year period using information from the Latin-American Collaborative Study of Congenital Malformations (ECLAMC) (10).

## MATERIALS AND METHODS

### Database description

The ECLAMC initiative is a multicenter international collaboration designed to identify associated risk factors and potential causes of congenital anomalies (CA). The data collection methodology has previously been reported (10). For the purpose of the present analyses, we followed a nested case-control design (10), analyzing information forwarded from each participating center to the ECLAMC headquarters. Retrospective review of data from the ECLAMC database encompassed information gathered between January 1989 and December 2012. We focused our evaluation on newborns diagnosed with hypospadias.

### Data collection and quality management

Data collection followed a standardized methodology for the entire study period.

Population: Briefly, each participating center conducted daily surveillance of all newborns looking for a detectable CA. For every detected case, the following information was collected: mother’s demographic data, prenatal and delivery information, and exposure to medications and toxic substances during pregnancy. Personnel trained specifically in the ECLAMC methodology at each institution conducted these assessments. For every enrolled case, the immediate next same-gender newborn was included as a control, collecting the same information.

Following approval of the study protocol by the ECLAMC board of directors and institutional ethics boards, information about all registered newborns with hypospadias and controls was gathered from the following countries: Argentina, Brazil, Bolivia, Chile, Colombia, Costa Rica, Ecuador, Paraguay, Peru, Uruguay and Venezuela. We excluded information from countries with incomplete registries, defined as those with more than 40% missing information in the database, and from those that failed to provide evidence of a continuous surveillance process over the study’s timeframe.

### Inclusion criteria

Isolated hypospadias (IH) cases were strictly defined as male newborns with an ectopic urethral meatus located along the ventral aspect of the penis and no other CA. Depending on location, these were further categorized as glanular, coronal, penile and scrotal. (Perineal and penoscrotal were included in this last category) (11). There were 29 megameatus intact prepuce variant cases and for that reason these patients were included in the glanular hypospadias group. Associated scrotal findings were also recorded. Newborns with associated anomalies were separately labeled as non-isolated hypospadias (NIH) cases. Each one of the associated anomalies was described in detail following the ECLAMC protocol for each of the different anomalies.

### Statistical analyses

Global prevalence analysis: For each center prevalence rates were registered annually during study period then aggregated and individualized (per country) hypospadias prevalence patterns over time were calculated, testing the null hypothesis of a zero slope curve as evidence of lack of increase over time. Annual rate changes were estimated from the slope of a regression line drawn through the observed values. The significance and linearity of the rate trend were tested following the Cochran and Armitage test to assess for gradient in proportions from several independent, quantitatively ordered samples (as reported by Fleiss) (12). This analysis was based on the formula:

Rate=Intercept + slope * year (based on y=b + mx; where m=slope, and b= intercept).

Exclusion of glanular cases prevalence analysis: To confirm the significance of the initial global trend we adjusted by hypospadias severity (exclusion of glanular cases) and performed a second analysis following a similar methodology as described before. We analyzed the population for trends, stratified based on country and hypospadias severity, as previously defined. In addition, this stratification was further evaluated for potential associations with NIH, reported as relative risks.

Secondary analysis adjusted for periods of time and countries: In order to give more support to the analysis, we verified our results and minimized biases by performing a secondary analysis for prevalence trends using a Poisson regression analysis, dividing the results for each country into periods of 5 years and adjusting for the effect of each hospital in the results. This was performed to reduce the effect of hospital registries in the results. The same analysis was performed after excluding all glanular cases for the reason mentioned above.

Associated anomalies analysis: For all NIH cases we analyzed the distribution of each associated abnormality and distribution based on hypospadias severity. In order to standardize assessment, we segregated associated anomalies by affected systems, i.e. genitourinary tract (GUT), gastrointestinal tract (GIT), limbs, facial anomalies (FA), cardiovascular (CV) and nervous system (CNS), abdominal wall (ABD), and others.

Lastly, we evaluated the impact of birth weight and maternal age in cases compared to controls using a Student T test. All analyses were conducted using Excel™.

## RESULTS

During the study period, a total of 192 centers in 12 countries supplied data to the centralized information center. After excluding countries with missing or incomplete information the final analysis comprised 159 hospitals from six South American countries.

**Figure 1:**
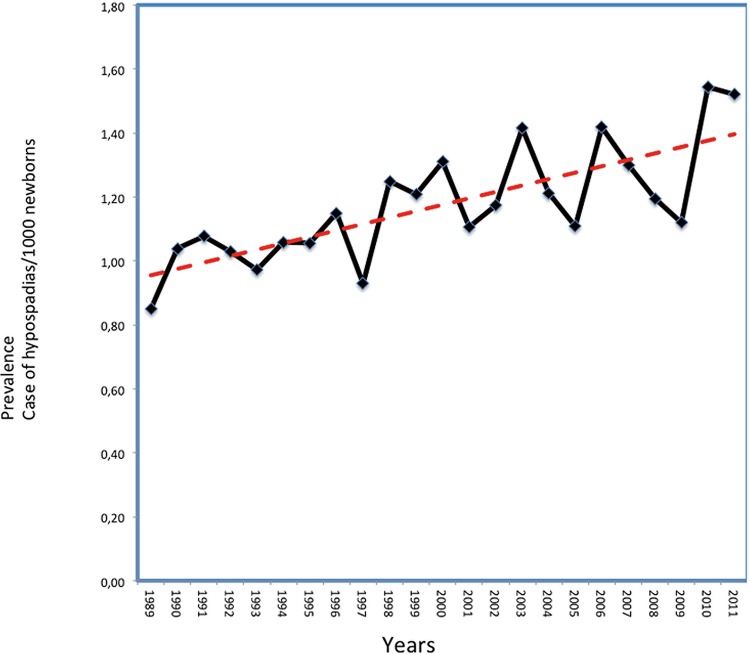
Global prevalence trend of patients diagnosed with hypospadias at birth in 6 different countries in South America. The increasing trend is statistically significant. (p<0.001).

Global population analysis: Between 1989 and 2013, the above-mentioned centers conducted surveillance on 4.020.384 newborns, detecting a total of 4.537 hypospadias, and resulting in a global prevalence of 11.3 per 10.000 newborns. Trend analysis demonstrated a global increase in annual prevalence of 0.2 hypospadias cases per 10.000 newborns per year (p<0.0001). This translates into a 3.3% increase over the study period (Figure-1).

The distribution of hypospadias cases by severity is shown in Table-1. A total of 82.2% of the hypospadias cases were isolated, with the remaining 17.8% being associated with other reported anomalies. Although we found an increase in prevalence trends for distal hypospadias, these failed to reach statistical significance (1.3% in 24 years).

**Table 1:** Distribution of hypospadias according to severity and association with other anomalies.

Type of Hypospadias	Total	Isolated	Non-Isolated	RR (95% CI)

	(N - %)^1^	(N)	(N - %)^2^	
Glanular	2189 (48.3%)	1822	376 (16.8%)	0.93(0.85–1.01)
Coronal	1800 (39.7%)	1521	279 (15.5%)	0.84(0.76– 0.94)
Penile	388 (8.6%)	286	102 (26.3%)	1.64 (1.33–2.03)
Scrotal	148 (3.4%)	96	52 (35.1%)	2.49 (1.80–3.47)

**Total**	**4534 (100%)**	**3725**	**809**	

^**1**^ = Percentage of each type of total; ^**2**^ = Percentage of Non-isolated cases of total cases in each type.

Analysis of the severity distribution and presence of associated anomalies during the entire study period revealed no association with distal cases (glanular hypospadias RR=0.93 [95% CI=0.85-1.01], coronal hypospadias RR=0.84 [95% CI=0.76-0.94]) whereas more proximal cases (penile hypospadias RR=1.64 [95% CI=1.33-2.03], scrotal hypospadias RR=2.49 [95% CI 1.80-3.47]) were significantly associated with other congenital anomalies.

When specifically evaluating NIH cases, we identified 809 patients with 1117 associated anomalies. On average there were 1.7 anomalies per NIH patient. The most common associated anomaly was cryptorchidism, representing 15.3% of associated anomalies, followed by minor facial anomalies (7.52%). Distribution analysis showed that after excluding minor facial anomalies, the most commonly affected system was the GUT (23.3%), followed by major FA (20.5%), CV (10%), CNS (8.7%), limbs (8.2%), GIT (6.3%), other anomalies (6%), and ABD (3.2%) Table-2. We detected 13 (1.6%) cases of Down’s syndrome associated with hypospadias (nine glanular, three coronal and two penile). There were eight (0.7%) cases associated with Edwards` syndrome. VACTERL association was diagnosed in five (0.4%) cases Table-2.

**Table 2:** Five most common associated anomalies by system and its distribution according to the severity of hypospadias.

Systems	Malformation	Glanular	Coronal	Penile	Scrotal	Total	Percent
Genito-urinary tract	Cryptorchidism	64	58	34	15	171	15.31
(23.8%)	Hydronephrosis	7	9	5	2	23	2.06
	Renal Agenesis	10	5	1	0	16	1.43
	Hydrocele	8	5	0	0	13	1.16
	Inguinal Hernia	7	4	1	1	13	1.16
Facial	Cleft lip/palate	33	31	5	8	77	6.89
(20.5%)	Micrognathia	22	21	7	4	54	4.83
	Low ear implantation	19	18	5	2	44	3.94
	Preauricular pit	20	17	3	0	40	3.58
	Microtia	5	8	1	0	14	1.25
Cardiovascular	Ventricular septal defect	16	9	1	6	32	2.86
(10.0%)	Single umbilical artery	7	10	4	4	25	2.24
	Auricular septal defect	9	6	3	2	20	1.79
	Valvular anomalies	5	2	5	0	12	1.07
	Patent Ductus arteriosus	1	3	3	0	7	0.63
Central Nervous system	Hydrocephalus	32	17	15	9	73	6.54
(8.7%)	Spina bifida	9	3	1	2	15	1.34
	Other	3	4	1	1	9	0.81
Limbs	Polydactyly	19	11	1	1	32	2.86
(8.2%)	Hip Displasia	8	7	1	0	16	1.43
	Clinodactyly	3	6	3	0	12	1.07
	Arthrogryposis	4	2	2	1	9	0.81
	Camptodactyly	3	1	0	0	4	0.36
Gastrointestinal tract	Imperforated Anus	13	12	13	3	41	3.67
(6.4%)	Esophageal atresia	7	5	3	2	17	1.52
	Duodenal atresia	2	3	1	0	6	0.54
	Anal stenosis	2	1	0	0	3	0.27
	Ileal stenosis	0	1	0	0	1	0.09
Abdominal wall defects	Omphalocele	9	10	0	1	20	1.79
(3.0%)	Diaphragmatic anomalies	0	4	1	1	6	0.54
	Rectus abdominus diastasis	1	3	1	1	6	0.54
	Gastroschisis	2	1	0	1	4	0.36
Others	Redundant skin at the neck	3	2	9	9	23	2.06
(6.0%)	Nevus	7	7	0	0	14	1.25
	Hemangioma	9	3	1	0	13	1.16
	Supernumerary nipple	3	6	1	0	10	0.90
	Pterigium colli	3	3	0	1	7	0.63

**TOTAL§**		**466**	**391**	**163**	**97**	**1117**	

§ Total number of cases includes cases not shown in table.

The average birth weight of hypospadias cases was 2914.2+/-621.6 grams, compared with 3251.1+/-753.6 for controls (p<0.001). These results were adjusted for gestational age. The average age of the mother at the time of delivery was 26.2 years old (SD+/-5.6y) for hypospadias cases and 26.3 years old (SD+/-7.4 y) for controls (p=0.27).

Secondary adjusted analysis: Chile, Brazil and Uruguay showed a statistically significant increase in prevalence, while Argentina, Colombia and Venezuela did not in the initial analysis. In the secondary analysis with grouping by periods of time, the Poisson regression showed no statistically significant increase or reduction in prevalence. Results per country showed a statistically significant increase in Uruguay and Venezuela in different periods of time during the study period Table-3. Uruguay showed the longest period of significant increase over time. On the other hand, Argentina was the only country with a trend towards reduction since 1992.

**Table 3:** Results from adjusted regression models evaluating temporal changes in the prevalence of total hypospadias from 1982 to 2011 in six countries of South America.

		Period					
		
Country ^1^		1982 - 1986	1987 - 1991	1992 - 1996	1997- 2001	2002 - 2006	2007 - 2011
ARG	IRR	1.0	0.93	0.75	0.84	0.74	0.60
	95%CI	(Ref.)	(0.74 – 1.16)	(0.62 – 0.92)	(0.71 – 1.00)	(0.58 – 0.95)	(0.37 – 0.98)
	P	-	0.501	0.005	0.055	0.016	0.039
BRZ	IRR	1.0	0.97	1.03	1.10	1.05	0.90
	95%CI	(Ref.)	(0.73 – 1.29	(0.72 – 1.48)	(0.79 – 1.54)	(0.79 – 1.38)	(0.66 – 1.22)
	P	-	0.830	0.871	0.583	0.750	0.489
CHL	IRR	1.0	0.86	0.88	0.76	0.89	0.96
	95%CI	(Ref.)	(0.52 – 1.41)	(0.54 – 1.44)	(0.58 – 1.00)	(0.64 – 1.23)	(0.73 – 1.25)
	P	-	0.545	0.617	0.054	0.476	0.770
COL	IRR	1.0	0.83	1.05	1.63	0.93	0.76
	95%CI	(Ref.)	(0.30 – 2.31)	(0.26 – 4.16)	(1.33 – 2.08)	(0.61 – 1.40)	(0.42 – 1.36)
	P	-	0.798	0.018	0.166	0.403	0.348
URU	IRR	1.0	1.37	1.15	1.55	1.67	1.92
	95%CI	(Ref.)	(0.92 – 2.04)	(0.65 – 2.06)	(1.09 – 2.19)	(0.98 – 2.85)	(1.16 – 3.18)
	P	-	0.120	0.627	0.015	0.058	0.011
VEN	IRR	1.0	1.16	1.67	1.01	1.12	1.58
	95%CI	(Ref.)	(0.88 – 1.52)	(1.30 – 2.14)	(0.74 – 1.37)	(1.04 – 1.19)	(1.49 – 1.67)
	P	-	0.294	<0.001	0.935	<0.001	<0.001
TOTAL	**IRR**	**1.0**	**0.98**	**0.99**	**1.02**	**0.99**	**0.93**
	95%CI	(Ref.)	(0.83 – 1.16)	(0.81 – 1.21)	(0.85 – 1.22)	(0.84 – 1.16)	(0.75 – 1.14)
	P	-	0.847	0.931	0.869	0.893	0.485

^1^ Countries: **ARG** = Argentina; **BOL** = Bolivia; **BRZ** = Brazil; **CHL** = Chile; **URU** = Uruguay; **VEN** = Venezuela; **IRR** = prevalence-rate ratios estimated from Poisson regression adjusted by hospital compared to the reference period; **95%CI** = 95% Confidence Interval; P value according the regression model; **ND** = no data coverage.

After excluding glanular cases, there was no significant increase or reduction in prevalence globally during the study period. Trends by country showed a reduction or increase in prevalence during different periods Table-4. The most significant changes were in Uruguay where an 80% reduction was recorded between 2002 and 2011.

**Table 4:** Number of total births, cases and rates (per 10,000 births) of severe hypospadias by period of time in six countries of South America.

		Period						
			
Country ^1^		1982 - 1986	1987 - 1991	1992 - 1996	1997- 2001	2002 - 2006	2007 - 2011	Total
ARG	Total births (N)	193.692	276.788	311.582	230.747	204.210	99.746	1,316.765
	Cases (N)^2^	98	118	123	113	93	39	584
	Rate^3^	5.1	4.3	3.9	4.9	4.6	3.9	4.4
	95% CI	(4.1 – 6.2)	(3.5 – 5.1)	(3.3 – 4.7)	(4.0 – 5.9)	(3.7 – 5.6)	(2.8 – 5.3)	(4.1 – 4.8)
BRZ	Total births (N)	235.217	204.756	193.227	161.080	185.003	130.900	1,110.183
	Cases (N)^2^	225	162	220	240	284	176	1.307
	Rat^e3^	9.6	7.9	11.4	14.9	14.8	13.2	11.7
	95% CI	(8.4 – 10.9)	(6.7 – 9.2)	(9.9 – 13.0)	(13.1 – 16.9)	(13.1 – 16.7)	(11.3 – 15.3)	(11.0 – 12.3)
CHL	Total births (N)	48.130	85.278	69.042	117.328	149.650	70.318	539.746
	Cases (N)^2^	31	38	45	59	56	20	249
	Rate3	6.4	4.5	6.5	5.0	3.7	2.7	4.6
	95% CI	(4.4 – 9.1)	(3.1 – 6.1)	(4.8 – 8.7)	(3.8 – 6.5)	(2.8 – 4.9)	(1.6 – 4.2)	(4.0 – 5.2)
COL	Total births (N)	11.672	14.762	9.571	2.286	48.308	33.116	119.715
	Cases (N^)2^	12	10	5	4	36	21	88
	Rate^3^	10.3	6.8	5.2	17.5	6.2	6.0	6.8
	95% CI	(5.3 – 18.0)	(3.2 – 12.5)	(1.7 – 12.1)	(4.8 – 44.8)	(4.2 – 8.9)	(3.7 – 9.3)	(5.4 – 8.4)
URU	Total births (N)	52.476	54.050	25.831	59.397	19.573	3.810	215.137
	Cases (N^)2^	23	24	4	21	6	1	79
	Rate^3^	4.3	4.4	1.5	3.5	3.1	2.6	3.7
	95% CI	(2.8 – 6.6)	(2.9 – 6.6)	(0.4 – 3.9)	(2.2 – 5.4)	(1.1 – 6.7)	(0.1 – 14.6)	(2.9 – 4.6)
VEN	Total births (N)	54.036	78.084	84.782	68.686	93.605	28.532	407.725
	Cases (N)^2^	34	33	44	22	45	40	218
	Rate^3^	6.3	4.2	5.2	3.2	4.8	14.0	5.3
	95% CI	(4.4 – 8.8)	(2.9 – 5.9)	(3.8 – 7.0)	(2.0 – 4.8)	(3.5 – 6.4)	(10.0 – 19.1)	(4.7 – 6.1)

^1^ Countries: **ARG** = Argentina; **BOL** = Bolivia; **BRZ** = Brazil; **CHL** = Chile; **URU** = Uruguay; **VEN** = Venezuela; **IRR** = prevalence-rate ratios estimated from Poisson regression adjusted by hospital compared to the reference period; **95%CI** = 95% Confidence Interval; P value aording the regression model.

## DISCUSSION

The present study provides epidemiological evidence that over the past two decades there has been an increase in hypospadias prevalence in different countries in South America and at different points in time. This finding is consistent with reports from other parts of the World. To our knowledge, our analysis is one of few large-scale studies to specifically focus on the Latin American population, and is particularly valuable given the limited information in our region (13). The global prevalence reported herein (11.3/10.000 newborns) is very similar to other studies worldwide (9, 14, 15). In agreement with data reported by Palouzzi et al., showing an increase in prevalence in the United States during a similar period of time, we also detected an increase in prevalence in South America. A similar trend was recently reported in Sweden (16).

Importantly, the ECLAMC database includes distal hypospadias cases in the analysis (15-17), this provides a more accurate picture of the true prevalence of the condition. Also, the methodology of a standardized physical examination on all newborns for every involved center reduces the probability of over-diagnosis. Surprisingly, in stark contrast with other studies such as the Metropolitan Atlanta Congenital Birth Defects Program (MACDP), we did not detect an increase in more severe hypospadias cases over time in the global analysis, and noticed that the trends favored higher prevalence at the expense of distal defects. Our detection procedure remained the same throughout the study confirming that these results are not a result of detection bias.

Other reports from developed countries have found an increase in prevalence of hypospadias of 1 to 4%, consistent with our increase of 3.3% (18). We acknowledge that an argument could be made for our findings being subject to artifact triggered by better reporting over time; however, the nature of our methodology dramatically reduced this potential bias. Nonetheless, the secondary analysis excluding glanular cases failed to support the initially detected increase. Specific regions showed an increase or reduction in prevalence. Given the latter there are important characteristics that deserve further evaluation, including the impact of industrialization, environmental pollution with different chemicals, and degree of urbanization in different regions with significant changes in prevalence. Our future studies will evaluate geographical and regional differences according to the secondary analysis results. It is interesting to highlight the different trends detected for different South American countries, raising the possibility of differences in socioeconomics and industrialization as an explanation for the reported trends. This hypothesis is supported by results from other studies, such as that of Li et al., who found that the global increase in prevalence in China was more evident in urban areas (19, 20).

The severity distribution in this report is consistent with previous literature, with a similar breakdown and predominance of distal hypospadias cases. Similarly, we corroborate literature that indicates an association between proximal hypospadias defects and other associated anomalies. For example, Leung et al. established that 20% of patients with hypospadias had other associated anomalies, similar to our results (20). Our data is novel in terms of exploring the degree of this association. For glanular and coronal hypospadias, we found no statistically significant results, whereas for penile and scrotal hypospadias we noted a positive association, with a gradient in favor of anomalies being more prevalent for the most proximal hypospadias defects. Some anomalies share similar molecular mechanisms that may explain their co-occurrence. A literature review on this topic provided little information. For instance, there are no reported associations between cleft lip/palate and hypospadias in the OMIM database, and many other studies have failed to detect links herein described between specific anomalies and proximal hypospadias. Although far from being conclusive and in clear need of further study, the information provided sets the basis for future studies specifically focusing on the presence of multiple anomalies in children with genitourinary congenital defects.

There are important shortcomings and limitations that deserve acknowledgement in the present study. We accept that the methodology employed is sub-optimal, as it did not include the entire population at risk (i.e. all male newborns in each country), leaving proportions of the population out of the analysis. Nevertheless, the surveillance protocol was strictly enforced, allowing us to capture information on a substantial number of babies over a long period of time. In addition, as previously mentioned, our findings may be driven by better detection compared to other surveillance systems, which conceptually can systematically favor good detection. Although plausible, the reported worldwide trends support the possibility of an increase in prevalence that has previously been reported for this large continental region but limited to specific regions for short periods of time.

Hospital-based sources of information may limit the final prevalence trend analysis due to differences in surveillance between centers during the study period. We tried to overcome this limitation with the secondary analysis. Lastly, it could be argued that the number of cases reported in this study could be biased due to detection in referral centers dealing with high-risk pregnancies. This, however, was not the case, as the included healthcare institutions were busy general hospitals.

Despite these perceived limitations, we propose that our results have value given the standardized way that the data was collected and that the study was conducted during the entire period. All included centers provided uninterrupted surveillance of their patients during their participation in the study, and centers with missing information were excluded to improve the quality of our results. Ultimately, this information supports prevalence patterns described in the literature but the secondary analysis made it difficult to conclude that there has been a clear increase in this previously under-evaluated part of the world.

## CONCLUSIONS

Our study is unique given the large geographical region, the study period, and sample size analyzed. We identified an increasing trend in hypospadias prevalence supported by a standardized methodology in specific regions and periods and novel information about associated congenital anomalies. However, subsequent analysis failed to find evidence supporting a global increase, particularly when severe hypospadias were selectively analyzed. Further studies are needed in order to determine whether environmental factors might be involved in specific regions.

## ABBREVIATIONS

ABD: Abdominal wall
CNS: Central nervous system
CV: Cardiovascular
ECLAMC: Latin-American Collaborative Study of Congenital Malformations
FA: Facial anomalies
GIT: Gastrointestinal tract
GUT: Genitourinary tract
IH: Isolated hypospadias
NIH: Non-isolated hypospadias
